# Health Care and Health Service Digital Revolution

**DOI:** 10.3390/ijerph17144913

**Published:** 2020-07-08

**Authors:** Roberto Lo Giudice, Fausto Famà

**Affiliations:** 1 Department of Clinical and Experimental Medicine, Messina University, AOU Policlinico “G.Martino”, Via Consolare Valeria, 98100 Messina, Italy; 2 Department of Human Pathology in Adulthood and Childhood “G. Barresi”, University Hospital “G. Martino” of Messina, Via Consolare Valeria 1, 98125 Messina, Italy; ffama@unime.it

**Keywords:** digital revolution, digital medicine, digital dentistry

## Abstract

The digital revolution has changed many aspects of the medical profession. Medical doctors and doctors in the dental sciences have been pushed to modify their workflow using new instruments such as decisional software, intraoral and extraoral scanners, and CAD-CAM technologies, which have improved diagnostics and the clinical/surgical phase of treatment and follow-up, and nowadays it is clear that medical professional life will continue in the era of digital medicine.

The digital revolution is a movement that has changed many habits in every aspect of our personal and professional life. As medical doctors and doctors in dental sciences, we have been pushed to radically change every aspect of our job, from diagnosis to prosthetic manufacturing.

Many new technologies that were once viewed as science fiction are now a reality, and clinicians are expected to make more precise diagnoses. Decisional software, using machine learning and data abstraction techniques, can correctly evaluate intra and extra-oral factors, linking them to patient compliance, to select the best treatment option among a variety of possible therapeutic plans, standardize every decision process, and provide diagnosis support to the clinician [1,2].

Moreover, this brand new prosthetic workflow has enabled clinicians to perform faster, more precise, and less annoying patient impressions [3,4]. In dentistry, the present-day generation of the intraoral scanner seems to be even more precise than traditional techniques of single and multiple crown rehabilitation on natural teeth. Many in vitro and vivo studies have reported on traditional impression (TI) and digital impression (DI) values of marginal and internal gaps, respectively, of 75.04 μm (SD = 13.12) (TI) and 55.01 μm (SD = 7.01) (DI) and 78.36 μm (SD = 19.66) (TI) and 59.20 μm (SD = 3.33) (DI), reaching a precision level that is more clinically relevant for long-term success [5].

The precision of digital impressions is evident; even full-arch impressions on implants with bar manufacturing show no statistically significant data (*p* > 0.05) in marginal bone loss with a 24-month follow-up [3].

The digital approach appears to be precise even for removable prosthesis rehabilitation, and when compared to irreversible hydrocolloid (ALG), polyvinyl siloxane (PVS), PVS modified with zinc oxide eugenol (PVSM), and an intraoral scanner (TRI) data, ALG (1.21 ± 0.35 mm), PVS (0.75 ± 0.17 mm; *p* = 0.008), PVSM (0.75 ± 0.19 mm; *p* = 0.012), and TRI (0.70 ± 0.18 mm; *p* = 0.006) display an accuracy that in superior to ALG and comparable to the other materials, leading to clinically acceptable results [6].

Moreover, the creation of prosthetic manufacturing has been changed using computer-aided design (CAD) and computer-aided manufacturing (CAM) technologies. Thanks to the flexibility of computer design and the precision of additive or subtractive manufacturing machines, the individualization of prostheses has reached a level of precision within a clinically acceptable range [7].

Considering that the digital revolution has provided an upgradable product to the clinician, frequent updates could correct some postprocessing errors and add new functions that could lead to a more precise final restoration in the future.

A revolution is a process that stretches from one era to another one; the digital revolution as a transition from the analog to the digital world is now over, and no one can ignore that its professional life will continue in the era of digital dentistry.
